# Transcriptome and phytochemical analyses provide insights into the organic sulfur pathway in *Allium hirtifolium*

**DOI:** 10.1038/s41598-020-80837-6

**Published:** 2021-01-12

**Authors:** Aboozar Soorni, Amir Mohammad Akrami, Reza Abolghasemi, Maryam Vahedi

**Affiliations:** 1 Department of Biotechnology, College of Agriculture, Isfahan University of Technology, Isfahan, 84156-83111 Iran; 2 Department of Horticulture, College of Agriculture, Isfahan University of Technology, Isfahan, 84156-83111 Iran; 3 Department of Horticultural Science, Faculty of Agricultural Sciences and Engineering, College of Agriculture and Natural Resources, University of Tehran, Karaj, Iran

**Keywords:** Plant biotechnology, Sequencing, Genetics, Plant sciences

## Abstract

*Allium* is one of the well-known genera of the Amaryllidaceae family, which contains over 780 species. Onions, garlic, leeks, and shallots are the most important species of this genus. *Allium hirtifolium* (shallot) is a rich source of proteins, carbohydrates, lipids, amino acids, and bioactive compounds such as organic sulfur compounds with an expansive range of biological activities and medicinal attributes. To identify the putative compounds and genes involved in the organic sulfur pathway, we applied GC–MS and RNA-seq techniques for the bulb, stem, and flower tissues of *A. hirtifolium.* The essential oil analysis revealed the maximum amount of sulfur compounds in stem against flower and bulb tissues. Transcriptome profiling showed 6155, 6494, and 4259 DEGs for bulb vs. flower, bulb vs. stem, and flower vs. stem, respectively. Overall, more genes were identified as being up-regulated rather than down-regulated in flower tissue compared to the stem and bulb tissues. Our findings in accordance with other results from different papers, suggest that carbohydrates are vital to bulb formation and development because a high number of identified DEGs (586 genes) were mapped to carbohydrate metabolism. This study has detected the genes in the organic sulfur pathway and indicated that the alliinase gene shows a high variability among different tissues. In general, this study formed a useful genomic resource data to explore tissue-specific sulfur pathway in *A. hirtifolium*, which is helpful for functional breeding.

## Introduction

*Allium* is one of the most prominent genera of the Amaryllidaceae family^1^, which contains over 780 species, mostly perennial plants with underground storage organs^2^. Onions, garlic, leeks, and shallots are the most important species of this genus^3^. Among them, *Allium hirtifolium* (shallot) is a delicious additive or condiment for foods and one of the edible and valuable medicinal plants, which is widely distributed in vast geographical areas of Iran^4^. This bulbous plant (called ‘‘Mooseer”) is endemic to Iran and was listed as an endangered species due to overexploitation, degradation of pastures, and pest infestations^4^. The organosulfur, phenolic, allicin, alliin, and ajoene are the most important and effective compounds in *A. hirtifolium,* which possess pharmacological roles due to antibacterial, antifungal, antiviral, antiprotozoal, and anthelmintic activities^5^. Alliin (S-alk(en)yl-l-cysteine sulphoxide) is the primary flavor precursor in the *Allium* genus, which rapidly degraded via the alliinase enzyme to produce allicin, pyruvate, ammonia, and a range of sulfur compounds^6^. As the chief biologically active constituent of sulfur-containing compounds in *Allium* species^7^, allicin facilitates defense response against pests and produces healthy compounds^8–11^.

RNA-seq is a powerful and efficient approach that has facilitated genes discovery related to sulfur metabolism, allergens and epitopes, identification, and characterization of alliinase isoforms, steroidal saponin pathway, and sucrose metabolism in different *Allium* species. Regarding the biosynthetic pathway of sulfur compounds, the transcriptome analysis of *A. sativum*^12^ discovered almost all of the genes involved in sulfur metabolism and two enzymes involved in glutathione biosynthesis. The primary organosulfur compound in *Allium* species is alliin, a natural substrate of alliinase^13^. Alliinase genes have been identified and sequenced in several *Allium* species. Previous results indicate variation in alliinase isoforms expression patterns among different tissues in different *Allium* species^14^. These data suggest the presence of two different isoforms, ISA1 and ISA2, which usually show high expression in the roots and bulbs without any expression in the leaves. *Allium* enjoys significant advantages but can induce food allergy symptoms through exposure to allergenic epitopes in garlic and onion sensitized individuals. Transcriptome dataset generated from onion bulbs has resulted in identifying putative genes accountable for allergenicity and different comparable sequences^15^.

Despite the fact that the pathway of synthesis of the organic sulfur flavor precursors has been deliberated with the assistance of transcriptome sequencing in some *Allium* species, however, this technique still can be performed for other species to acquire more knowledge of secondary metabolites biosynthesis, different possible routes for the synthesis of the major flavor precursor, and alliinase genes variation. Enriching our understanding of *A. hirtifolium* genetics and secondary metabolic pathways will be led to the development of new resources for the functional breeding and conservation of this species. Hence, in this study, the genes expressed in different tissues of *A. hirtifolium* were studied using a transcript pair-end sequencing strategy to identify where individual genes show high or low expression levels. The transcriptome was annotated and also analyzed the pathway of sulfur and essential genes such as Alliinase. In the current report, genes related to the sulfur pathway, alliinase genes, and the presence of different isoforms in the *A. hirtifolium* have been discovered for the first time with a novel report for Iranian endemic species.

## Results

### Identification of volatile organic compounds

The GC/MS analysis identified a total of 16 sulfide compounds in all three different tissues. Table 1 shows the results of the qualitative and quantitative essential oil analyses. As shown, 1-butene, 1-(methylthio)-(Z), methyl methylthiomethyl disulfide, and Dimethyl tetrasulfide were the most abundant compounds, which comprised 19.72–33.96% of the essential oil in different tissues. On the other hand, several compounds such as 2,4-dithiapentane, 4-mercaptopyridine, and methyl n-butyl disulfide were present with the lowest percentages. In general, all compositions were dominant in aerial parts compared to the bulb, with the most quantity of sulfur compounds found in the stem against the flower and bulb tissues.

**Table 1:** List of important organosulfur compounds identified in essential oil of *A. hirtifolium.*

	RI (retention index)	Flower (area %) ± SD	Stem (area %) ± SD	Bulb (area %) ± SD
1-Butene, 1-(methylthio)-(Z)	< 900	15.31 ± 0.01	17.21 ± 0.01	9.11 ± 0.01
Methyl methylthiomethyl disulfide	< 900	8.21 ± 0.01	9.43 ± 0.01	6.23 ± 0.01
Dimethyl tetrasulfide	< 900	6.93 ± 0.01	7.32 ± 0.01	5.08 ± 0.01
Dipropyl disulfide	< 900	6.11 ± 0.01	6.91 ± 0.01	4.71 ± 0.01
Diisopropyl trisulfide	< 900	4.87 ± 0.01	5.87 ± 0.01	3.13 ± 0.01
5-Chloroorcylaldehyde	< 900	4.28 ± 0.01	5.33 ± 0.01	3.22 ± 0.01
Dimethyl disulfide	< 900	3.67 ± 0.01	4.21 ± 0.01	2.98 ± 0.01
Dimethyl trisulfide	< 900	3.54 ± 0.01	4.13 ± 0.01	2.87 ± 0.01
Piperitenone oxide	< 900	2.89 ± 0.01	3.23 ± 0.01	2.13 ± 0.01
2,3,5-Trithiahexane	< 900	2.43 ± 0.01	2.87 ± 0.01	1.51 ± 0.01
Chloromethyl methyl sulfide	< 900	1.99 ± 0.01	2.67 ± 0.01	1.45 ± 0.01
N-butylBenzene sulfonamide	< 900	1.76 ± 0.01	2.11 ± 0.01	1.55 ± 0.01
2,3,4-Trithiapentane	< 900	1.29 ± 0.01	2.69 ± 0.01	1.39 ± 0.01
Methyl n-butyl disulfide	< 900	1.12 ± 0.01	1.99 ± 0.01	1.32 ± 0.01
4-Mercaptopyridine	< 900	1.10 ± 0.01	1.65 ± 0.01	1.25 ± 0.01
2,4-Dithiapentane	< 900	1.01 ± 0.01	1.28 ± 0.01	1.11 ± 0.01

### RNA-seq and de novo assembly

RNA-seq of nine libraries from flower, stem, and root tissues resulted in 263.8 million reads that less than 4% and 10% not exhibited a quality score of Q20 and Q30, respectively. After trimming and eliminating short and poor quality reads, more than 80.2% of reads (211.72 million reads) remained for the assembly (Table 2).

**Table 2:** Descriptive statistics of reads quality and quantitative before and after trimming.

Tissue sample	Replicate	Total bases	Read count	GC (%)	Q20 (%)	Q30 (%)
**Original data**						
Flower	1	4,830,021,598	31,986,898	42.55	96.68	91.11
Flower	2	4,038,774,350	26,746,850	42.39	96.39	90.43
Flower	3	4,288,224,150	28,124,750	42.33	96.33	90.66
Stem	1	4,454,515,100	29,500,100	42.84	96.45	90.53
Stem	2	4,286,079,734	28,384,634	43.07	96.53	90.72
Stem	3	4,365,244,114	28,455,544	43.17	96.13	90.33
Bulb	1	5,162,667,350	34,189,850	43.31	96.93	91.63
Bulb	2	4,105,387,094	27,187,994	43.31	96.44	90.62
Bulb	3	4,334,177,124	29,227,564	43.32	96.22	90.72
**Clean data**						
Flower	1	3,867,279,209	26,097,930	42	98.15	93.5
Flower	2	3,129,715,192	21,160,290	42	97.95	92.95
Flower	3	3,387,284,100	23,360,580	42	97.95	92.95
Stem	1	3,572,809,929	24,151,324	42	98.01	93
Stem	2	3,315,190,505	22,401,330	42	98.05	93.14
Stem	3	3,496,752,504	23,787,432	42	98.06	93.1
Bulb	1	3,990,018,602	26,873,072	43	98.29	93.83
Bulb	2	3,070,080,093	20,898,896	43	98.06	93.2
Bulb	3	3,311,103,456	22,993,774	43	98.06	93.24

Clean reads obtained from samples were independently assembled de novo using Trinity and rnaSPAdes, and the resultant assemblies were analyzed to evaluate quality. Based on the evaluation metrics performed, the comprehensive comparison showed that Trinity achieved the best results with a longer assembly size and N50, and the lowest number of contigs. Hence, the Trinity assembly was selected as the best for conducting downstream analyses. By using Trinity, clean reads were assembled into 172,190 genes and 373,862 transcripts, with a total assembled length of 115.211 and 289.476 Mbp for genes and transcripts, respectively. The N50 value and mean length of genes were 959 and 669 bp, while these values for transcripts were 1065 and 774.29 bp, respectively (Table 3). Comparing the EvidentialGene sets with the Trinity and rnaSPAdes assemblies indicated that the tr2aacds pipeline achieved better Trinity output results. The tr2aacds pipeline reduced the Trinity transcript number by 2.5 fold about 226,625, increased the N50 value, and produced longer genes and transcripts. The results of the percentage of reads that were mapped back to the final assembly, ranging from 90.71 to 92.84%, demonstrated that the tr2aacds pipeline is capable of generating higher-quality transcripts by removing redundant or combining the high-quality transcripts. The tr2aacds showed a better performance in assembly completeness between two assemblies, as evaluated by the BUSCO dataset (the Eukaryota lineage). According to the BUSCO results, 91% and 4.7% contigs were complete and missing in both assemblies, respectively, while the percentage of duplicated in *tr2aacds* pipeline reduced from 47 to 20% (Fig. S1).

**Table 3:** Summary stats report of transcriptome assemblies obtained using Trinity, rnaSPAdes, and tr2aacds.

Trinity			rnaSPAdes
	Transcript	Gene	–
Total	373,862	172,190	210,123
Average contig	774.29	669.1	623.2
Total assembled bases (Mbp)	289.476	115.21	256.67
Contig N50	1065	959	875
**tr2aacds pipeline from the EvidentialGene package**			
Total	147,237	92,343	176,349
Average contig	833.74	854.71	734.6
Total assembled bases (Mbp)	122.757	78.926	102.76
Contig N50	1105	1214	987

### Differentially expressed genes

The replicates’ correlation results (*r* = 0.94–0.96) and the biological coefficient of variation (Disp = 0.01076, BCV = 0.1037) indicated that the estimated gene expression values are incredibly consistent between any replicate pair of each tissue, and samples are appropriately separated.

Based on the EdgeR-based analysis, a total of 10,155 DEGs were identified among the three comparisons of bulb vs. flower (BF), bulb vs. stem (BS), and flower vs. stem (FS). Among DEGs, 6155 genes were found to be significantly differentially expressed in BF. Of these, expression of 2132 and 4023 genes were up-regulated in bulb and flower, respectively. The EdgeR-based analysis identified 6494 DEGs in BS, including 2776 and 3718 up-regulated genes in bulb and stem, whereas 4259 DEGs were detected in FS with a total of 2968 and 1291 up-regulated genes in flower and stem, respectively. Furthermore, 479 DEGs were common to the three comparisons of BF, BS, and FS.

### TFs identification

Since TFs genes are involved in regulating various physiological systems and play multiple key roles in plants by controlling the synthesis of bioactive components, we performed a detailed analysis of deferential TFs to provide further insights into the complex molecular mechanisms involved in the organic sulfur metabolism.

**Figure 1:**
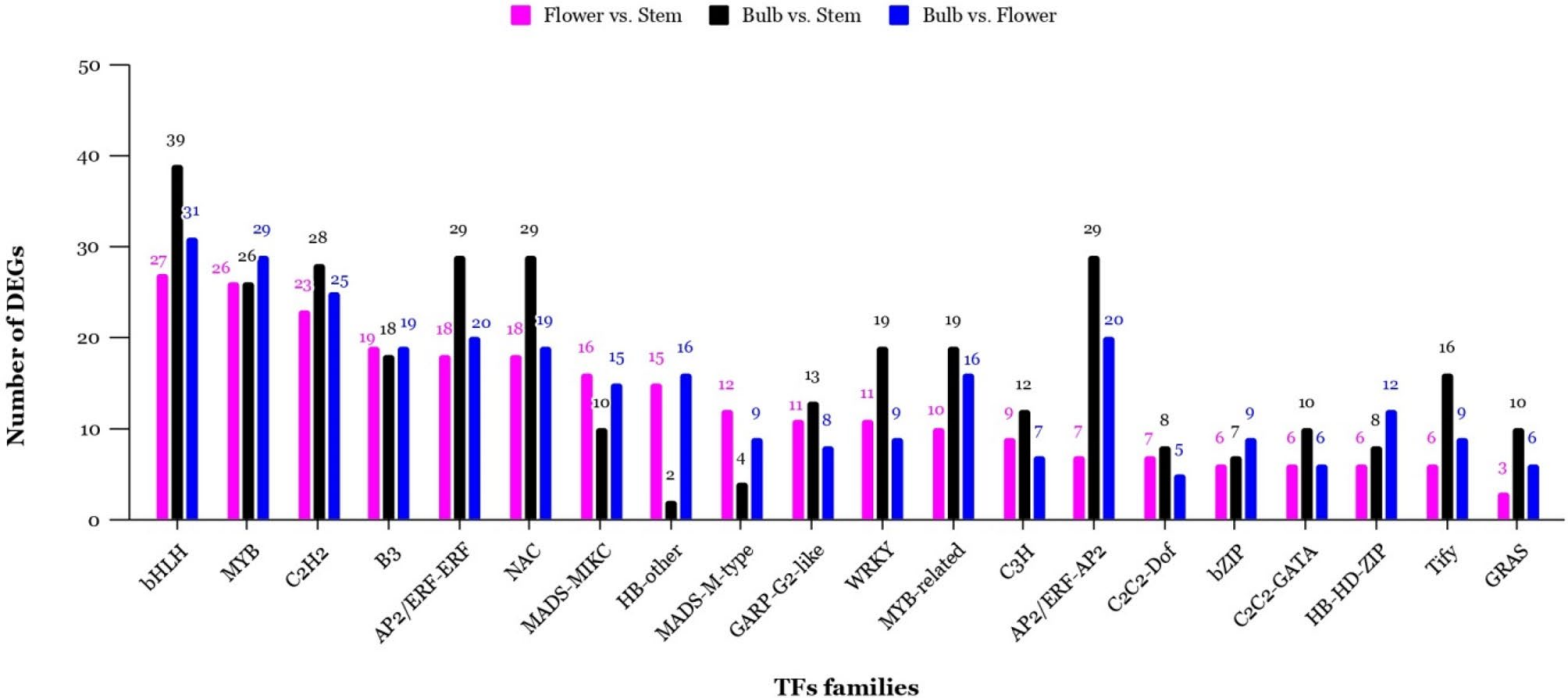
Top 20 families of differentially expressed transcription factors in three main comparison of flower vs. stem, bulb vs. stem, and bulb vs. flower.

From 10,155 DEGs, 625 transcripts, representing 6.15% of the total DEGs, were annotated to 58 transcription factor (TF) families. Among these TF families, bHLH (56) was the most abundant, followed by MYB (48), AP2/ERF-ERF (42), C2H2 (41), NAC (36), B3 (31), WRKY (28). Figure 1 summarizes the number of DEGs belonging to the top 10 TF families in three pairwise comparisons (Fig. 1).

### Enrichment analyses of gene ontology (GO) annotations and KEGG pathways

**Figure 2:**
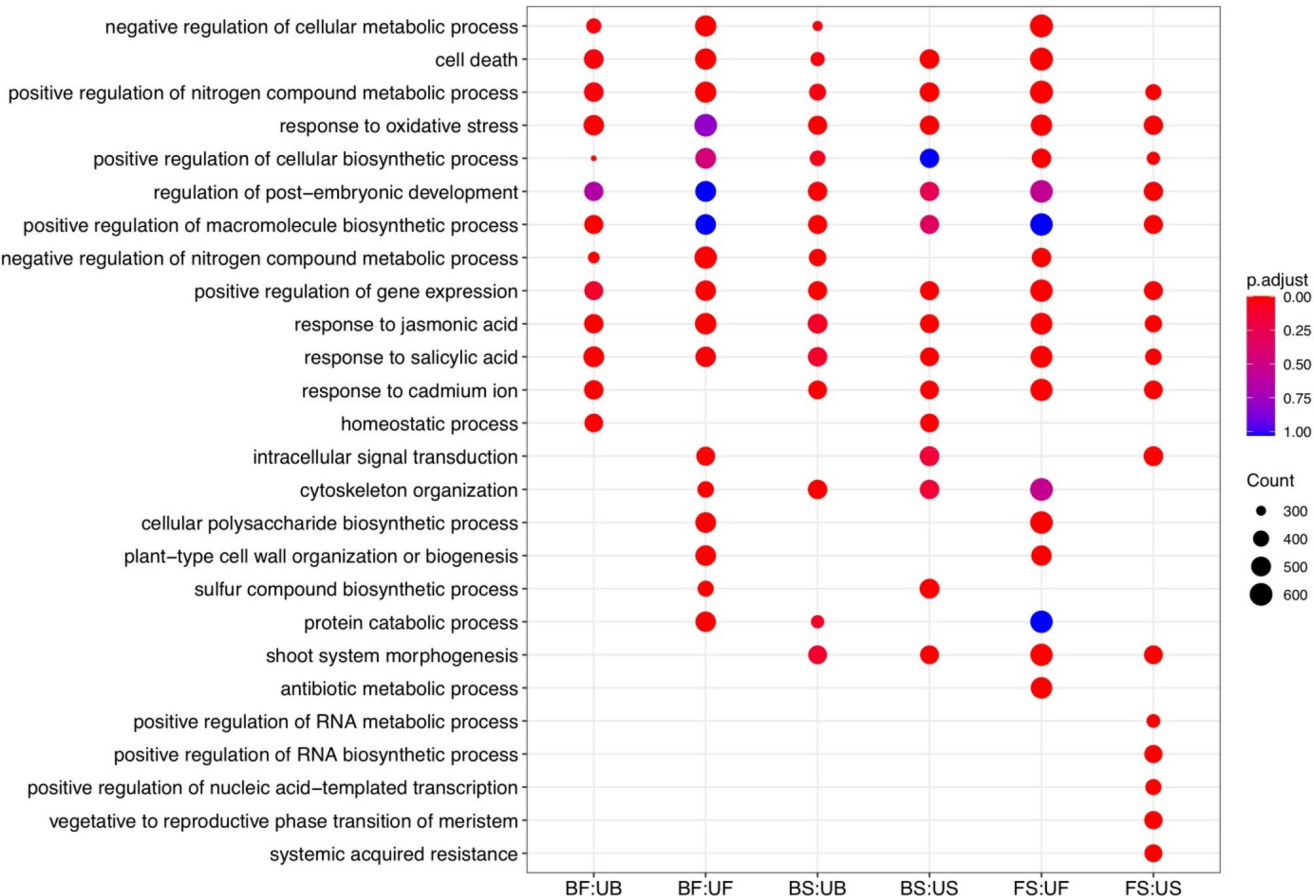
Biological process ontology of the differentially expressed genes identified among tissues comparison. F, S, and B represent flower, stem and bulb tissues, respectively. Here, U represents up-regulated term.

**Figure 3:**
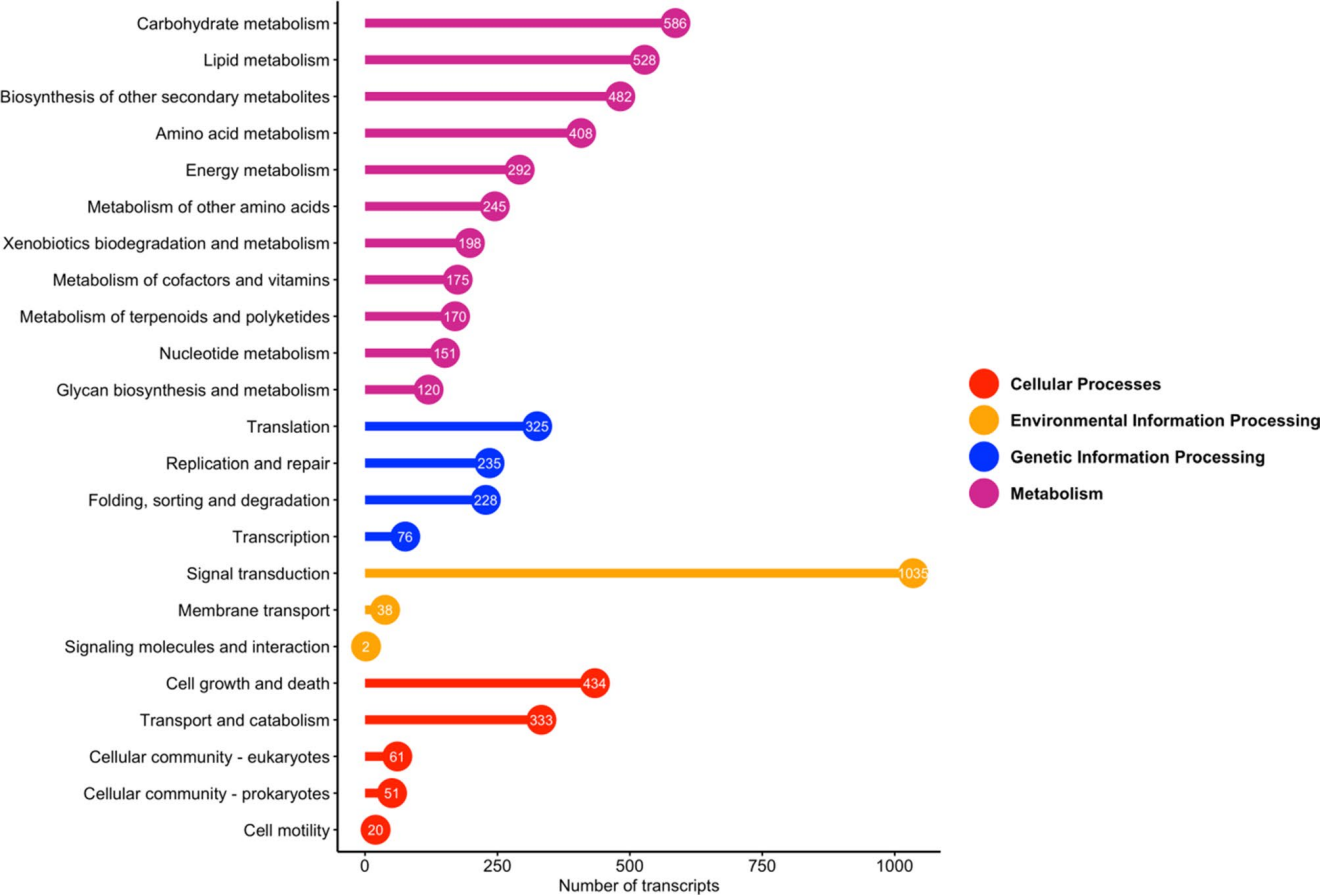
Overview of KEGG pathway maps of DEGs defined by KAAS. Bars represent the number of assignments of unigenes to each term.

To demonstrate valuable information and further explore the possible roles of the DEGs, GO and KEGG enrichment analyses were conducted. Among the 10,155 DEGs selected to predict functions by GO annotation, 9465 DEGs were assigned to at least one GO term classified into 63 groups, including 31 biological processes, 12 molecular functions, and 20 cellular components (Table S1). Next, GO enrichment analysis was carried out on each set of identified DEGs in tissues comparison (Fig. 2). The GO enrichment analysis of DEGs identified several unique or common biological processes at different tissues, indicating that different genes play important roles in developing *A. hirtifolium*. Over-representation analysis of biological functions revealed “sulfur compound biosynthetic process” and “nitrogen compound metabolic process” terms that were expected to be significantly enriched in the flower and stem. Interestingly, GO term enrichment analysis among the flower DEGs showed the biological process category of organic substance metabolic process, primary metabolic process, nitrogen compound metabolic process, and metabolic cellular process were mostly enriched GO terms under metabolic process subcategory. In contrast, the organonitrogen compound metabolic process and phosphorus metabolic process were the most abundant terms in the bulb DEGs (Fig. S2). According to the participating KEGG metabolic pathway, the KEGG analysis divided 7868 DEGs into four branches (Fig. 3): metabolism (3355 DEGs), environmental information processing (1075 DEGs), cellular processes (899 DEGs), and genetic information processing (864 DEGs). The significant pathways containing more than 500 DEGs were “signal transduction” (1035, 13.15%) followed by “carbohydrate metabolism” (586, 7.44%) and “lipid metabolism” (528, 6.71%). Additionally, we performed KEGG pathway enrichment analysis on the genes up-regulated in different tissues (Fig. S3). The results revealed that the significantly enriched pathways involved starch and sucrose metabolism, ribosome, MAPK signaling pathway, and plant hormone signal transduction. It is noteworthy that 12 of the up-regulated genes in flower were enriched in glutathione metabolism, participated in the organic sulfur metabolism.

### Putative pathway for organic sulfur biosynthesis

**Figure 4:**
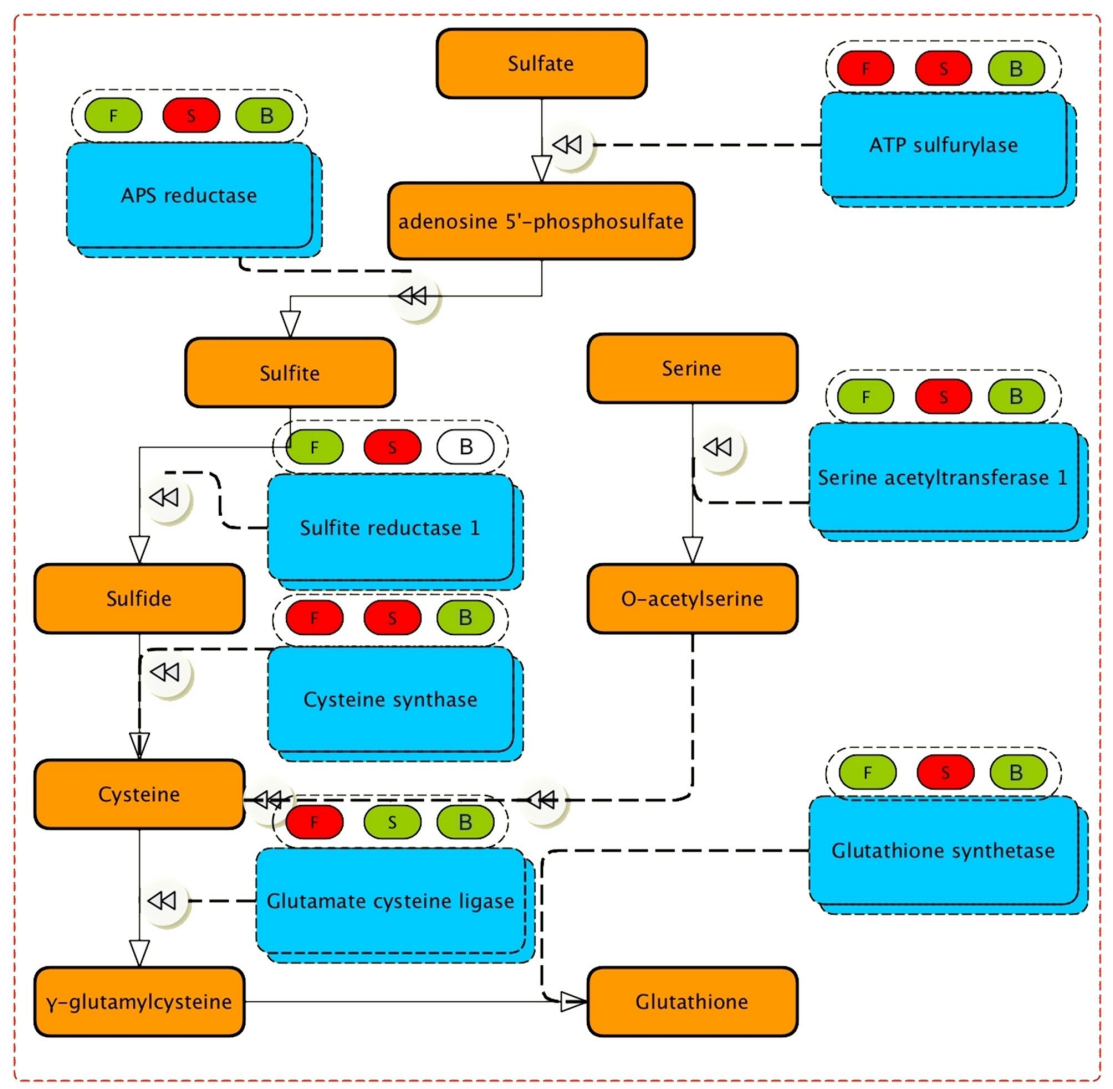
Expression patterns of unigenes involved in the organic sulfur pathway. F, S, and B represent flower, stem, and bulb tissues, respectively. The red and green circles represent up-regulated and down-regulated DEGs, respectively.

Seven genes involved in the organic sulfur metabolism and glutathione biosynthesis (Fig. 4) existed in the transcriptome data sets of *A. hirtifolium*. Overall, all genes were identified as being up-regulated in aerial parts. Four unigenes, including APS reductase, sulfite reductase, serine acetyltransferase, and glutathione synthetase, showed significantly increased expression in the stem. In contrast, glutamate-cysteine ligase was found to be up-regulated only in flowers. ATP sulfurylase and cysteine synthase were up-regulated in both stem and flower tissue samples against the bulb.

**Figure 5:**
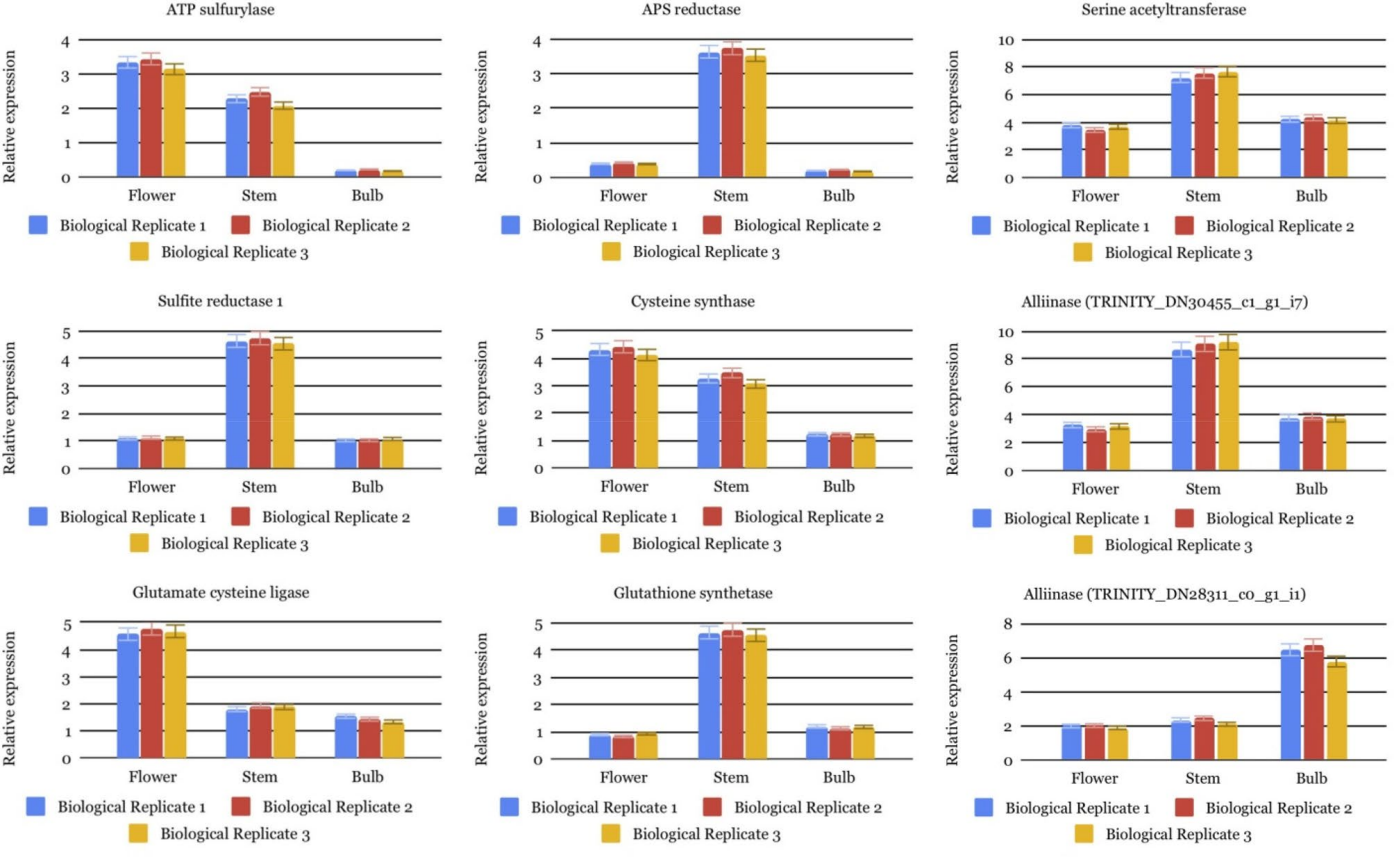
Confirmation of the transcriptome sequencing data by quantitative real-time (qRT)-PCR. Nine differentially expressed genes (DEGs) involved in sulfur pathway.

To evaluate transcriptome results and validate the expression levels of DEGs involved in the organic sulfur pathway, qRT-PCR was performed for all genes identified in this pathway. Expression trends were consistent for all genes in qRT-PCR (Fig. 5) and RNA-seq analyses. The outcomes confirmed that the expression patterns of qRT-PCR were pretty matched with the results of RNA-seq data so that all the chosen genes were determined to be most abundant in the stem and then in the flower. The achieved consequences of qRT-PCR indicated that the transcriptomic profiling data estimated from RNA-seq were pretty reliable. The correlation between the RNA-seq and qRT-PCR measurements was 0.93 (r^2^ = 0.93, Fig. S4).

### Alliinase gene family and phylogenetic relationships

**Figure 6:**
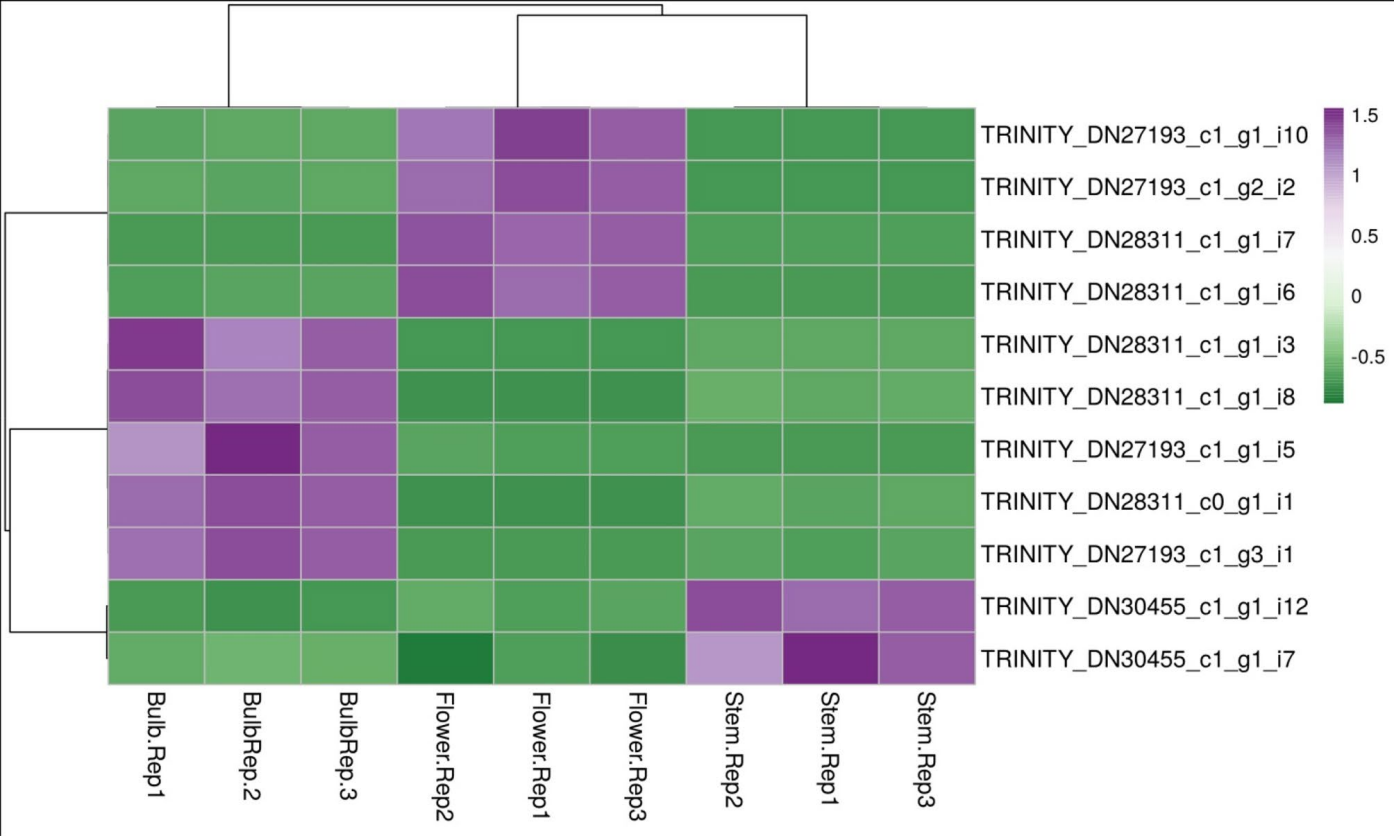
Heatmap of differentially expressed *Alliinase* genes family.

In this study, a fragment of approximately 1091 bp of four distinct alliinase genes was successfully found in *A. hirtifolium,* which showed dissimilar patterns of expression in different tissue samples. These data suggested that “TRINITY_DN30455_c1_g1” gene (Fig. 6) with two isoforms had a high expression in the stem compared to flower and bulb, while isoforms expression patterns belonging to other identified genes were variable between flower and bulb tissues. The presence of various isoforms is a possible explanation for the difference in the amount of allicin among different tissues.

The phylogenetic tree constructed from the alliinase genes belong to different *Allium* species revealed that alliinase genes from *A*. *hirtifolium*, *A*. *macrostemo, and A. tuberosum* were grouped into one cluster, while *A*. *cepa* and *A*. *sativum* were classified into other clusters (Fig. S5).

**Figure 7:**
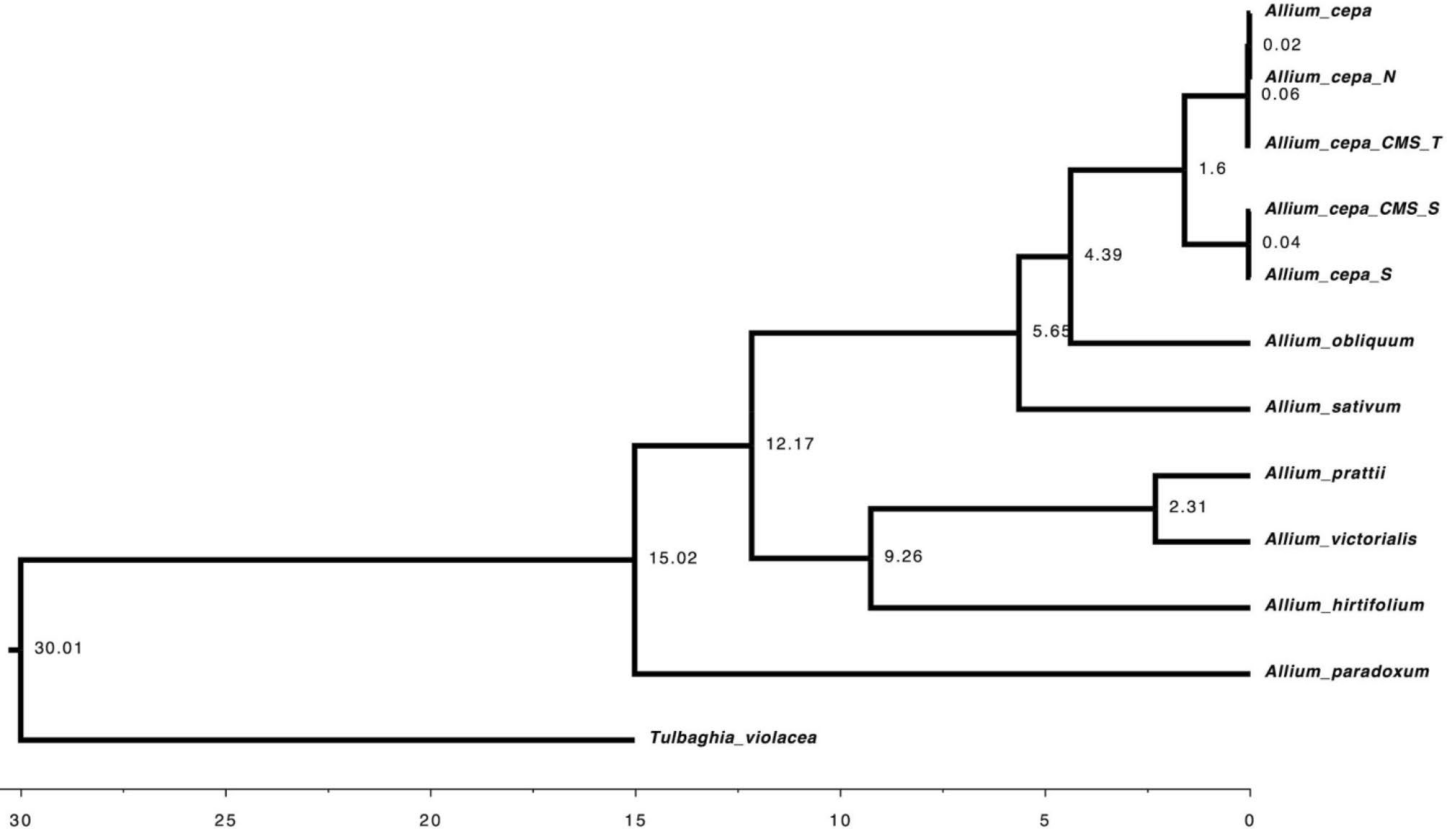
Phylogenetic relationships among *Allium* species inferred from cpDNA data.

In terms of the number, 35 genes (Table S2) belong to eight different species were collected to construct the phylogenetic tree using the BEAST analysis. The phylogenetic tree derived from the cpDNA data (Fig. 7) indicated that the split between the *A*. *hirtifolium* and *A. cepa* grouped with *A. sativum* has occurred ~ 12.17 Mya. As the phylogenetic tree yielded, *A*. *hirtifolium* was not placed in the same clade or group with *A. cepa* and *A. sativum*. Hence, *A*. *hirtifolium* could likely be considered as one of the ancestors of *A. cepa* and *A. sativum*.

## Discussion

The most *Allium* species have been valued for their flavors and health-giving benefits arising from organic sulfur compounds^16,17^. *A. hirtifolium* is one of the edible, precious, and valuable medicinal plants of the *Allium* genus, widely distributed in extensive geographical areas of Iran^4^. It is a rich source of secondary metabolites, particularly disulfide and trisulfide (sulfur) compounds^3^. Although allicin is the most important organosulfur compound in *Allium* species, research works have reported other sulfur compounds from the essential oil of these species with important beneficial effects and pharmacological roles. Few works have additionally been stated about the composition of the essential oil of *A. hirtifolium*^18,19^. Still, our work was the first evaluation of volatile sulfur compounds in different tissues of this species. Fasihzadeh et al. identified four volatile sulfur compounds including 1-butene,1-(methylthio)-(Z) (18.21%), methyl methylthiomethyl disulfide (8.41%), dimethyl tetrasulfide (6.47%), piperitenone oxide (4.55%) as the most plentiful components in the essential oil of *A. hirtifolium*^18^, which is in agreement with the outcomes of the current study. Besides dimethyl disulfide, dimethyl trisulfide, 2,3,5-trithiahexane, chloromethyl methyl sulfide, and N-butylBenzene sulfonamide, which were the most dominate sulfur compounds identified in tissue culture organs of *A. hirtifolium*^19^, Dipropyl disulfide, and Diisopropyl trisulfide were found to be abundant in the current study. Mahboubi et al. also presented 5-chloroorcylaldehyde as one of the significant sulfides in *A. hirtifolium* oil^20^, consistent with the results obtained in this study. Among *Allium* species, organic sulfur synthesis has a similar pathway, but secondary metabolite biosynthesis and accumulation are related to regulator genes with tissue-specific expression patterns, which highly affect the quantities and chemical compositions in each *Allium* species^21^. Given this, RNA-seq has been applied effectively to discover genes related to sulfur metabolism in some *Allium* species such as *A. cepa*^14,22,23^, *A. sativum*^12,24^, and *Allium fistulosum*^25^. So, the transcriptome analysis in another species allows us to provide a more in-depth exploration of various aspects of the sulfur pathway. It can also improve the accuracy of related genes expression in different tissues. On the other hand, the expression dynamics of unigenes and their transcriptional profiles, especially for DEGs involved in the organic sulfurs synthesis pathways, could speed up the engineering of secondary metabolites in this species. Hence, different tissues of *A. hirtifolium*, including flower, stem, and bulb, were selected for transcriptome analysis to explain the sulfur pathway, gene families related to allicin biosynthesis, and evaluation of phylogenetic relationships.

Despite the large genome size in *Allium* species, *A. cepa* and *A. sativum* with almost the same genome size (~ 17 Gb)^26^, the high quality of the reads obtained from sequencing and transcriptome assembly metrics indicated assembled transcriptome is highly accurate and reliable for downstream analysis. Primary assembly using Trinity assembler produced a total of 373,862 transcripts. In contrast, the tr2aacds pipeline belongs to EvidentialGene set could generate a secondary assembly with 147,236 transcripts (122.757 Mbp) and an N50 value of 1105 bp, indicating a better de novo assembly of transcriptome compared to previous studies with *Allium* species such as *A. cepa*^15^, *A. sativum*^12^, and *A*. *fistulosum*^25^.

In this way, a large number of differentially expressed unigenes (10,155 genes) were revealed among three tissues by transcriptome analyses, which were investigated to TFs identification, GO classification, and clarify their role in the secondary metabolite pathways. Among them, more genes had been observed be differentially expressed between bulb and stem (6494 genes) followed by bulb and flower comparison (6155 genes) than between pair of stem and flower tissues (4259 genes), which probably indicating, more genes are involved in the growth and development of bulbs. The organic sulfur pathway is the well-characterized pathway among *Allium* species, and TFs are the key roles for regulating this pathway. Our findings indicated bHLH, MYB, AP2/ERF-ERF, AP2/ERF-AP2, C2H2, and NAC are highly represented among DEGs, suggesting their potential functions in regulating organic sulfur biosynthesis. Additionally, transcriptome analysis indicates MYB transcription factors control diverse biological processes such as regulating primary/secondary metabolism and hormone syntheses. In contrast, NAC family members participate in regulating plant growth and developmental processes^27,28^. Although the previous studies have revealed TFs are associated with secondary metabolism pathways^29,30^, little is about the TFs involved in the biosynthetic pathway of sulfur compounds. However, previous research has demonstrated that MYB, NAC, WRKY, and AP2/ERF are the most represented TFs in the transcriptome data of root, stem, bulb, and leaves of different *Allium* species^31^.

According to the GO classifications analysis, organic substance metabolic and phosphorus metabolic were the most important prominent processes, very similar to what has been reported in previous research related to *Allium* species. It demonstrates *A. hirtifolium* has a distinguishing feature in the biosynthesis of secondary metabolites, especially the metabolic network of organic sulfur compounds. The GO enrichment analysis unveiled that “sulfur compound biosynthetic process” and “nitrogen compound metabolic process” were highly enriched in stem and flower, which suggested that they play a crucial role during *A. hirtifolium* development and the synthesis and metabolism of organic sulfur compounds. Subsequently, the graph of the KEGG category’s enrichment analysis showed a high number of DEGs mapped to terms in the metabolism group. Interestingly, a high number of identified DEGs (586 genes) were mapped to carbohydrate metabolism, followed by 528 transcripts to lipid metabolism, similar results in the *Allium* searches^12,25^. Previous studies indicate that about 65% of bulb dry-matter in onion and garlic consists of glucose, fructose, and sucrose as the main carbohydrate components^32,33^, evidence for the essential role of genes involved in carbohydrate metabolism for the formation and development of *A. hirtifolium* bulbs. This analysis allows the detection of most of the paralogous genes and helps to gain insight into the carbohydrate metabolism principles in bulb's emergence and improvement at the molecular level. In the KEGG enrichment analysis, “starch and sucrose metabolism” constituted the primary metabolism pathway. All of the researches in the literature support the view that sucrose metabolism is essential for bulb development in bulbous ornamentals^34^. KEGG enrichment results showed that the DEGs were also highly enriched in “plant hormone signal transduction pathway”, “MAPK signaling pathway”, and “phenylpropanoid biosynthesis pathway” especially in flowers, suggesting that these pathways may be closely related to the organic sulfur metabolism in *A. hirtifolium*.

Furthermore, the investigations presented in the current study identified most genes involved in the organic sulfur metabolism and glutathione biosynthesis for the first time in *A. hirtifolium.* In this pathway, seven genes were identified as being up-regulated in aerial parts. The high content of sulfur compounds in fresh leaves and aerial parts of *A. cepa*^13^, *A. sativum*^35^, and *A. hirtifolium*^36^ may explain that the genes associated with the sulfur pathway have tissue-specific expression patterns and are mostly active in aerial parts. This result was validated by quantitative real-time PCR analysis so that similar observations have been made. In *Allium* species, the non-protein sulfur amino acids are hydrolyzed by the enzyme alliinase to produce flavor compounds^3^. Alliinase is a critical enzyme that operates in the allicin biosynthesis pathway^6^ and plays some roles in the plant defense mechanism against pathogenic microbes and herbivores^11,37^. Alliinase is encoded by a gene family^38,39^ and finds in all garlic tissues, onion, and other *Allium* species^23,40–45^. Hence, this study also focused on identifying of alliinase genes and their expression patterns in different tissue samples of *A. hirtifolium.* Functional transcriptome analysis revealed 11 alliinase transcripts belonging to four distinct genes expressed in all three tissues with a different pattern. It has been previously detected and demonstrated^14^ that the isoforms expression pattern of alliinase gene is variable among different tissues of various *Allium* species. Previous studies^14,44^ have also described the presence of nonhomologous alliinase genes in different tissues of *A*llium species, which is consistent with the results obtained in this study.

Finally, a phylogenetic tree was constructed using the cpDNA data to broaden our knowledge about the phylogenetic relationship of *A. hirtifolium* with other *Allium* species. According to the phylogenetic tree, *A. obliquum* was supported to be the sister of *A. cepa*^46^, and *A. hirtifolium* was closely clustered with *A. victorialis* and *A. prattii,* and perhaps an ancestor of garlic and onion.

## Conclusions

In the current study, we used the GC–MS technique to identify the putative compounds of essential oil and generated a fully annotated transcriptome assembly for flower, stem, and bulb tissues of *A. hirtifolium*. Phytochemical analysis reveals 1-butene, 1-(methylthio)-(Z) as the main sulfur component of *A. hirtifolium* essential oil. In particular, the transcriptome of *A. hirtifolium* contained the genes involved in the organic sulfur pathway. Of these unigenes, the alliinase gene indicated high variability in expression patterns among different tissues. The results also showed that many genes regulate carbohydrate metabolism, which is essential for the formation and development of *A. hirtifolium* bulbs. Additionally, bHLH, MYB, AP2/ERF-ERF, and C2H2 constituted a large number of DEGs encoding TFs in all tissues, suggesting their potential functions in regulating organic sulfur biosynthesis. Our finding represents a valuable initial resource with a purpose to enable further research on the molecular mechanisms of sulfur biosynthesis and studies of the functional genomics and molecular genetics of this valuable medicinal herb.

## Materials and methods

### Sample collection, RNA extraction, and sequencing

**Figure 8:**
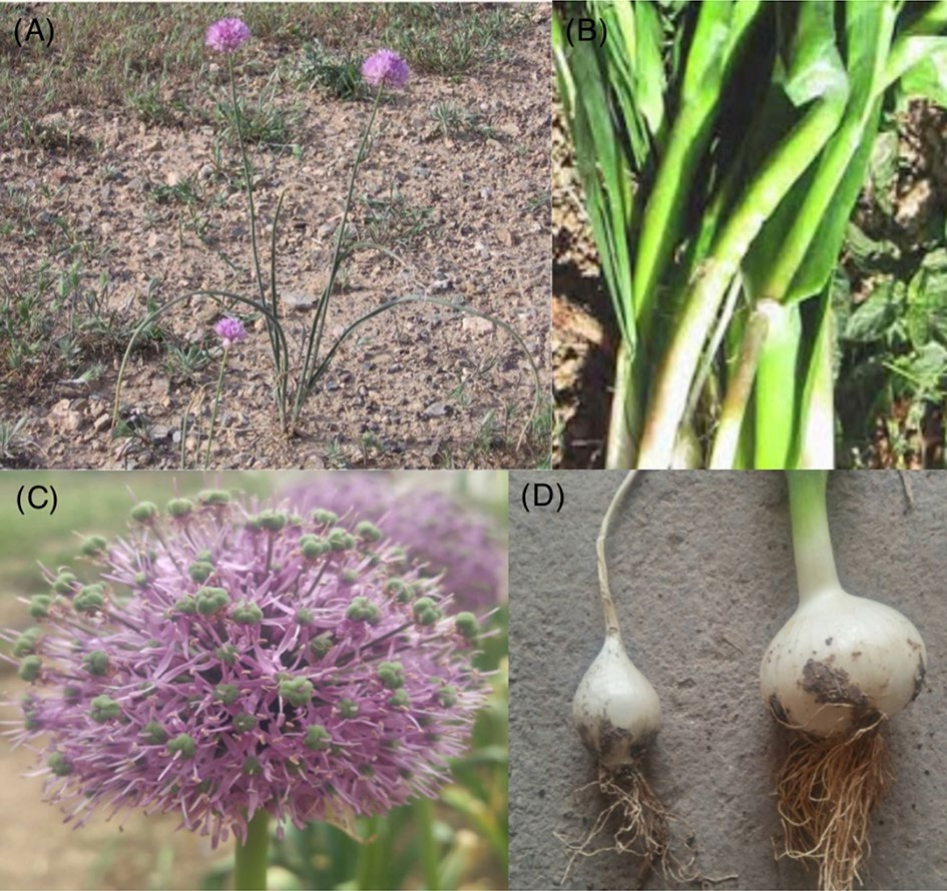
Tissues of *A. hirtifolium* used for de novo transcriptome assembly. (**A**) *A. hirtifolium* growth habit in nature; (**B**) stem; (**C**) flower; (**D**) and bulb.

Plant tissue samples of 15 individuals’ *A. hirtifolium* were collected from the same population in April 2018 from the mountainous region of Fereydunshahr with an altitude of 2365 m above sea level (32.951512° N and 50.112014° E), Iran. The permissions were not necessary to collect these samples. The formal identification of the plant material was undertaken by the herbarium of Agricultural and Natural Resources College, University of Tehran, and no voucher specimens were collected and deposited in the collection (it is not necessary as we do not describe a novel species). Plant parts, including flowers, stems, and bulbs (Fig. 8), were stored in a liquid nitrogen dewar and returned to the lab frozen until further processing. To extract RNA, we designed a study on three biological replicates created from five pooled samples for each tissue (thus, five plants were used for each biological replicate of each tissue) to decrease the variance caused by interindividual differences in gene expression. The TRIzol Reagent (Thermo Fisher Scientific, USA), combined with Qiagen RNAeasy Columns (QIAGEN company, Germany), was used to separate and isolate RNA from the tissue samples. The cDNA libraries were constructed according to the instructions given in TruSeq Stranded mRNA LT Sample Prep Kit (illumina, USA). They then were subjected to sequencing on an Illumina HiSeq 2000 paired-end 151 bp system at the Beijing Genomics Institute (BGI)-Shenzhen, Shenzhen, China (http://www.genomics.cn/index.php). With three biological replicates, altogether, nine samples were used for sequencing in this study. All RNA-seq data were deposited in the NCBI SRA database under the project PRJNA628019.

### Volatile sulfur compounds analysis

For gas chromatography–mass spectrophotometry (GC/MS) investigation, bulb, stem, and flower tissues were dried at room temperature (25 °C). At this point, isolation of the volatile compounds was carried out for 1 g of each sample by utilizing a manual Solid Phase Microextraction (SPME) fiber with the layer of carboxen/polydimethylsiloxane (CAR/PDMS)^47^. GC examination was done on the Thermoquest-Finnigan gas chromatograph instrument (Thermo Fisher Scientific). It was equipped with a Flame ionization detector (FID) and contained a DB-5 capillary column with 30 m long, 0.25 mm internal diameter, and 0.25 μm stationary phase film thicknesses. The column's thermal programming started at an initial temperature of 60 °C and ramping up till 250 °C at 5 °C/min. The injector's temperature and the detector were set to 250 °C and 280 °C, respectively. The GC–MS analysis was carried out on the Thermoquest-Finnigan Trace instrument with the identical prerequisites described for GC analysis. The helium carrier gas had a constant flow rate of 1 ml/min, and the ionization energy was 70 eV. The volatile compounds were identified by comparing their retention indices with available authentic samples and literature information (The Pherobase database; http://www.pherobase.com/) and comparing the mass spectra of each essential oil constituents with mass spectra databases available in GC–MS libraries. Identification was also determined using co-injection with standards. The relative percentages of each compound were calculated according to the area under its curve in the GC apparatus's chromatogram spectrum.

### Data filtering and de novo assembly

After sequencing, we performed a high-level quality check for each of our samples by using FastQC (https://www.bioinformatics.babraham.ac.uk/projects/fastqc/) program. Subsequently, the Trimmomatic v0.30 program^48^ was utilized to clear out poor quality reads and bases, with parameters included Illumina clip with seed mismatches 2, palindrome clip threshold 30 and simple clip threshold 10 to cut adapter and other illumina-specific sequences from the reads, leading and trailing quality three to remove low quality or N bases from the earliest starting point and last part of reads, sliding window trimming with a window size four and an average quality 30 inside the window, and read length of 50 bp. Then, de novo transcriptome assembly was constructed and evaluated with Trinity^49^ and rnaSPAdes^50^ programs to assemble clean reads. The transcriptome assemblies were further subject to the EvidentialGene tr2aacds pipeline (http://eugenes.org/EvidentialGene/) to eliminate redundant transcripts and acquire a ‘most efficient’ set of de novo assembled transcripts. Benchmarking Universal Single-Copy Orthologs (BUSCO) v4^51^ was implemented with default settings to estimate transcriptome completeness using the eukaryote conserved gene datasets.

### Digital expression profile analysis

The RNA-seq by Expectation Maximization (RSEM) method^52^ with the bowtie2 parameters was used to quantify the gene expression level. Clean reads of each library were mapped back onto the assembled transcriptome, then read count from all samples were combined into a matrix using script abundance_estimates_to_matrix.pl. After producing the reads count matrix, pearson’s correlation coefficient between each pair of technical replicates was evaluated by comparing the log_10_ of FPKM values. Finally, the differentially expressed genes (DEGs) were analyzed using the R Bioconductor package, edgeR^53^. Both statistical significance thresholds, including “FDR ≤ 0.001 and the absolute value of Log2 fold change (Log2FC) ≥ 4” were simultaneously applied to detect consistently differentially expressed genes. Functional annotation of differentially expressed genes (DEGs) was conducted using BLASTX v2.2.29 and BLASTP v2.2.29 to search against the Swissprot-Uniprot database^54^. To explore transcription factors (TFs) in *A. hirtifolium* transcriptome, DEGs sequences were compared to the iTAK database using the default parameters^55^. The gene ontology (GO) for the transcripts was assigned using the Kyoto Encyclopedia of Genes and Genomes (KEGG^56^ maps, while the WEGO (http://wego.genomics.org.cn^57^; software was utilized to attain GO functional classification. Using the ClusterProfiler R package^58^, we performed GO and KEGG pathway enrichment analyses to identify the main biological processes and metabolic pathways in DEGs. The GO terms and pathways with *q* value (adjusted p-value by BH method) < 0.05 were considered to be significantly enriched ones.

### Validation of DEGs using quantitative real-time PCR analysis

Quantitative real-time polymerase chain reaction (qRT-PCR) was employed using the SYBR Green PCR Master Mix (Takara, Dalian, China) following the manufacturer’s instructions at an ABI ViiA 7 Real-time PCR platform with a total reaction volume of 20 μL, containing 10 μL of SYBR GreenMaster, 2 μL of diluted cDNA template, 1 μL of each primer (10 μM), and 7 μL of water to examine the reliability of RNA-seq results. The qRT-PCR analyses were performed for nine genes involved in the organic sulfur pathway, and their expression profiles were compared within flower, stem, and bulb samples with three biological and technical replicates. Gene-specific primers were designed using the primer designing tools of IDTdna (http://www.idtdna.com). The qRT-PCR conditions were set as standard conditions: 95 °C for 3 min then followed by 40 cycles of 95 °C for 10 s, 60 °C for 20 s, 72 °C for 20 s, and finally completed with a melting curve program. The gene expression was normalized using *Actin* as a reference gene, and the relative expression levels were calculated using the 2^−∆∆Ct^ method^59^.

### Comparative phylogenetic analysis

To determine the relationship between the identified alliinase gene in *A. hirtifolium* and the sequences downloaded from the databases, a multiple alignment was run using MUSCLE v3.8.31^60^. Then, the maximum likelihood method in RaxML^61^ was done for phylogenetic tree construction. To investigate phylogenetic relationships of *Allium* species and estimate divergence time, we used chloroplast data (cpDNA) to construct a phylogenetic tree. In this regard, chloroplast data were collected from the assembly sequences of *A. hirtifolium* using a homology search against the chloroplast genome of *A. cepa*. The resulting sequences were multiply aligned using MUSCLE v3.8.31^60^. Output files were further subjected to the trimAl v1.4^62^ with the parameter “− gt 0.8 − st 0.001” to eliminate the poorly aligned regions. Subsequently, a bayesian analysis using Markov chain Monte Carlo (MCMC) implemented in BEAST v.2.5.2^63^ was used to reconstruct the phylogenetic tree and estimate divergence time according to the HKY model with four gamma categories and strict molecular clock parameters. The calibration dates were obtained from TimeTree (http://timetree.org). The trees were then interpreted by the program TreeAnnotator v1.6.1 before generating a maximum clade credibility tree. The obtained tree was visualized and edited using FigTree v1.4.0 program (http://tree.bio.ed.ac.uk/software/figtree/).

## Supplementary Information

Supplementary Information 1.

Supplementary Information 2.
