# Supplementary material for: Transcriptome and phytochemical analyses provide insights into the organic sulfur pathway in Allium hirtifolium
Source: Sci Rep. 2021 Jan 12;11:768. doi: 10.1038/s41598-020-80837-6 (PMC7804154; doi:10.1038/s41598-020-80837-6)
Supplement: Supplementary file 1 — Supplementary Information 1. [file 41598_2020_80837_MOESM1_ESM.docx]

**Transcriptome and phytochemical analyses provide insights into the organic sulfur pathway in *Allium hirtifolium***

## Aboozar Soorni^1*^, Amir Mohammad Akrami^1^, Reza Abolghasemi^2^, Maryam Vahedi^3^

^1^ Department of Biotechnology, College of Agriculture, Isfahan University of Technology, Isfahan 84156-83111, Iran

^2^ Department of Horticulture, College of Agriculture, Isfahan University of Technology, Isfahan 84156-83111, Iran

^3^Department of Horticultural Science, Faculty of Agricultural Sciences and Engineering, College of Agriculture and Natural Resources, University of Tehran, Karaj, Iran

Corresponding Author:

Aboozar Soorni

Email address: [**soorni@iut.ac.ir**](mailto:soorni@iut.ac.ir)


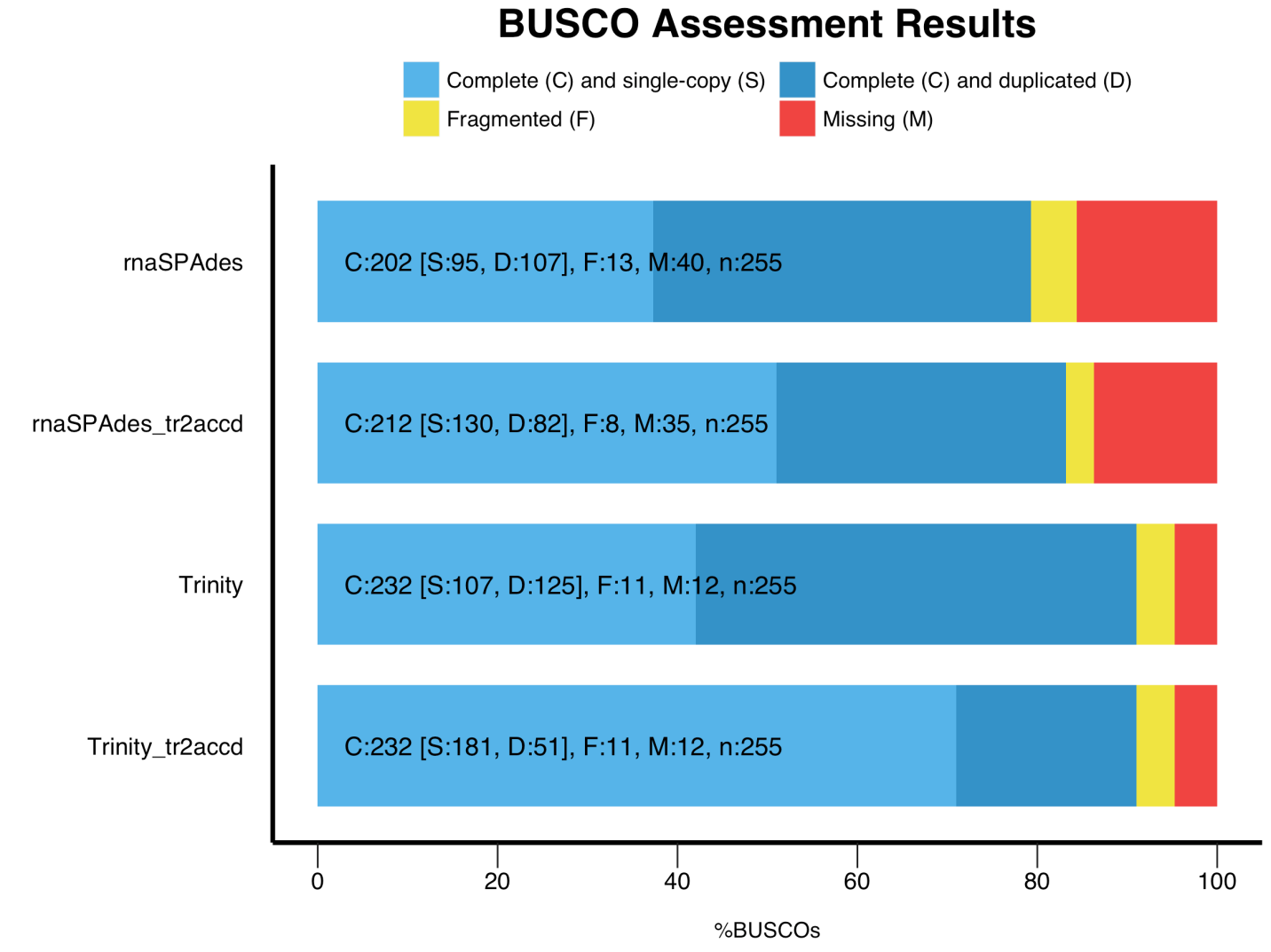


Figure S1. The completeness assessment results of the Trinity, rnaSPAdes, and tr2accd transcriptome assemblies of *A. hirtifolium* using BUSCO. Colors refer to the percentage of the complete single-copy orthologs, complete and duplicated orthologs, and fragmented ortholog


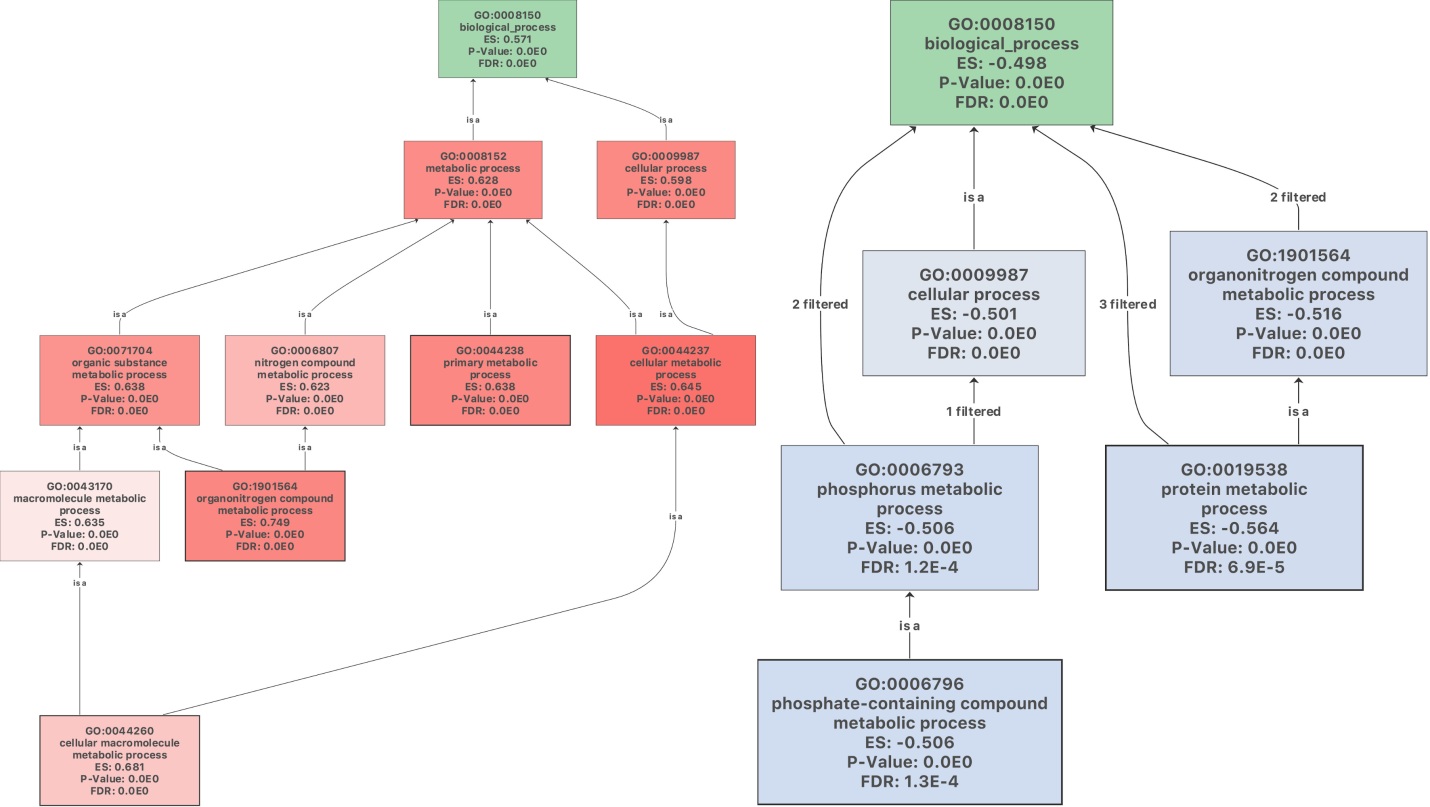


Figure S2. GO enrichment analysis of differentially expressed genes (DEGs) related to the biological process.


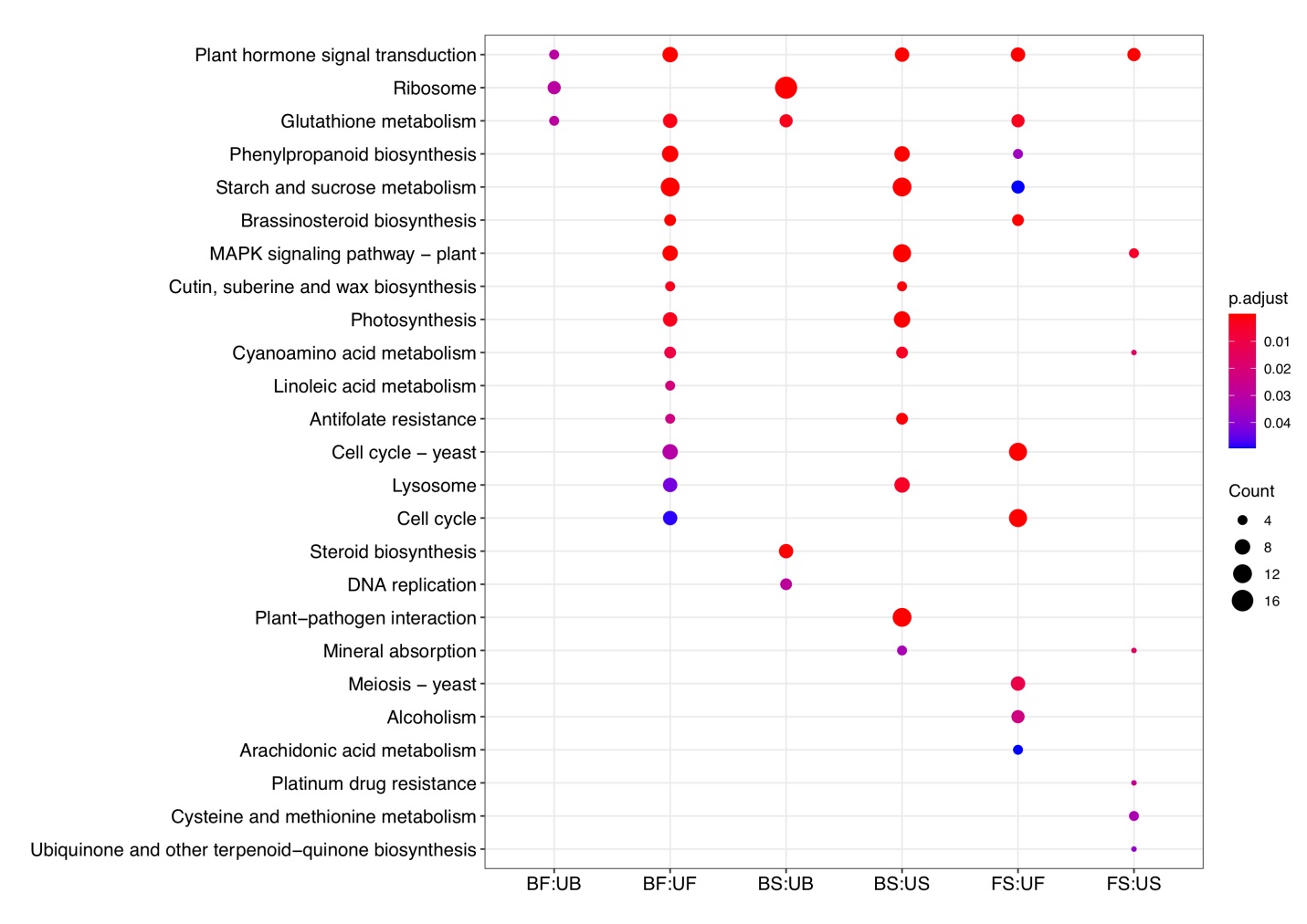


Figure S3. KEGG pathway enrichment analysis of DEGs.


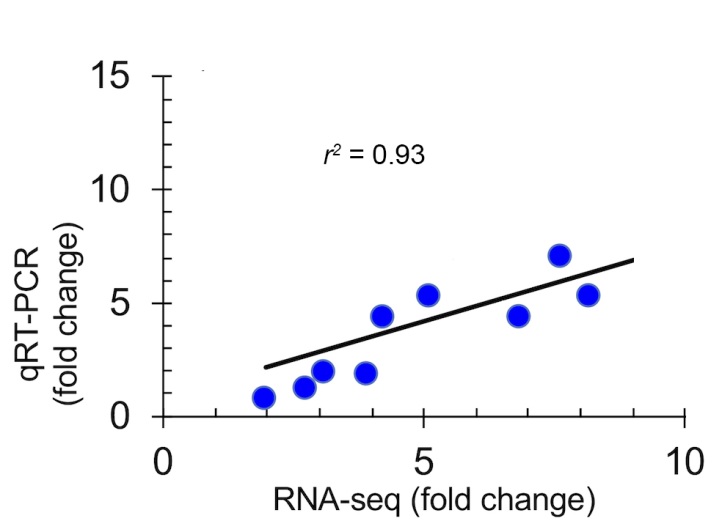


Figure S4. Pearson correlation between fold changes in genes expression determined by qRT-PCR and RNA-seq.


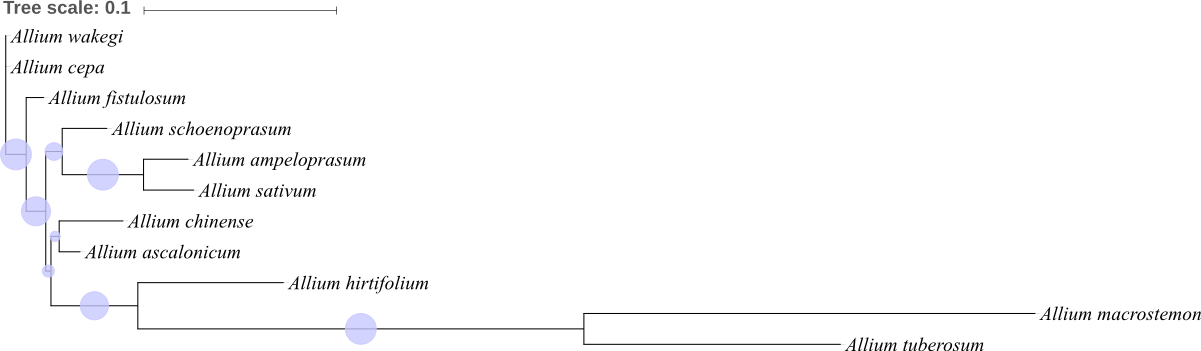


Figure S5. Phylogenetic analysis of *Allium* species using alliinase gene through the maximum likelihood method.

Table S1. Summary of GO classification analysis in differentially expressed genes

| GO Summary | | | | | |
| --- | --- | --- | --- | --- | --- |
|  |  | bulb vs. Flower | bulb vs. Stem | Flower vs. Stem | Total |
|  |  |  |  |  |  |
| Annotated Genes | | 5,706 | 5,989 | 4,300 | 9,465 |
| GO Terms | Biological Process | 4,495 | 4,837 | 3,324 | 7,547 |
|  | Cellular Component | 4,693 | 4,989 | 3,622 | 7,914 |
|  | Molecular Function | 4,923 | 5,229 | 3,695 | 8,204 |

Table S2. Name and sequence length of chloroplast genes extracted from transcriptome data of *A*. *hirtifolium*

| Gene | Length | Gene | Length | Gene | Length | Gene | Length |
| --- | --- | --- | --- | --- | --- | --- | --- |
| accD | 1229 | psaB | 2204 | rbcL | 1438 | rps18 | 305 |
| atpA | 1417 | psaC | 245 | rpl14 | 368 | rps19 | 125 |
| atpB | 1496 | psbA | 662 | rpl20 | 353 | rps3 | 662 |
| atpF | 554 | psbB | 1526 | rpl33 | 200 | rps4 | 605 |
| atpI | 741 | psbC | 1417 | rpl36 | 113 | ycf1 | 1417 |
| ccsA | 783 | psbD | 1061 | rpoA | 985 | ycf2 | 1526 |
| clpP | 113 | psbE | 251 | rpoB | 1358 | ycf3 | 505 |
| matK | 231 | psbI | 110 | rpoC1 | 1584 | ycf4 | 554 |
| psaA | 1276 | psbK | 185 | rpoC2 | 2235 | - | - |
